# Polymorphism of *SMAD7* gene (rs2337104) and risk of colorectal cancer in an Iranian population: a case-control study 

**Published:** 2014

**Authors:** Zahra Akbari, Nahid Safari-Alighiarloo, Mohammad Yaghoob Taleghani, Farzaneh Sadat Mirfakhar, Hamid Asadzadeh Aghdaei, Mohsen Vahedi, Atena Irani Shemirani, Ehsan Nazemalhosseini-Mojarad, Mohammad Reza Zali

**Affiliations:** 1 *Gastroenterology and Liver Diseases Research Center, Shahid Beheshti University of Medical Sciences, Tehran, Iran*; 2 *Proteomics Research Center, Faculty of Paramedical Sciences, Shahid Beheshti University of Medical Sciences, Tehran, Iran*; 3 *Basic and Molecular Epidemiology of Gastrointestinal Disorders Research Center, Shahid Beheshti University of Medical Sciences, Tehran, Iran*

**Keywords:** *SMAD7*, Colorectal cancer, Single nucleotide polymorphism (SNP)

## Abstract

**Aim:** The purpose of this study was to evaluate the influence of intronic polymorphism of the *SMAD7* (Mothers Against Decantaplegic Homolog 7) gene (rs2337104) on the risk of colorectal cancer (CRC) and clinicopathological features in an Iranian population.

**Background:** *SMAD7* has been identified as an antagonist of transforming growth factor beta (TGF-b)-mediating fibrosis, carcinogenesis, and inflammation. Regarding to the recent genome-wide scan, a risk locus for colorectal cancer at *18q21* has been found, which maps to the *SMAD7* gene.

**Patients and methods:** This case-control study was performed on 109 CRC patients and 109 healthy controls recruited in Taleghani Hospital. The genotyping of all samples were done by TaqMan assay via an ABI 7500 Real Time PCR System (Applied Biosystems) with DNA from peripheral blood. The association of this polymorphism with the risk of CRC and clinicopathological features was investigated.

**Results**: Our results indicated that there were no significant association between genotypic and allelic frequencies of *SMAD7* polymorphism (rs2337104) and CRC risk in our population. Although the T allele is the most frequent one in this population and its frequency was 86.7% in patients compared with 91.7% in controls (OR=1.705, 95% CI= 0.916–3.172). Also, the *SMAD7* genotypes were not associated with any clinicopathological characteristics in CRC patients (P>0.05).

**Conclusion:** For the first time, this study results revealed that this *SMAD7* polymorphism couldn’t be a potential risk factor for CRC or a prognostic biomarker for prediction of clinicopathological features in an Iranian population. A large-scale case-control study is needed to validate our results.

## Introduction

 One million people are diagnosed with colorectal cancer (CRC) worldwide each year (1). It is the third most common cancer leading the fourth cause of worldwide cancer mortality (2,3). Regarding, CRC is one of the common diseases in an Asian population (4); an increased incidence has been reported in Iran during three last decades (-). It has been estimated that genetic factors cause over 30% of the variation in colorectal cancer susceptibility and majority of this is originated from multiple low risk mutations (8).

The transforming growth factor beta (TGF-b) signaling pathway has a pivotal role in cancer initiation and progression. It acts as tumor suppressor in the early stages of tumorigenesis and as pro-oncogene of cancer progression and metastasis in more advanced stages of epithelial tumors (9). Identification of components' variants of this pathway may reveal a good marker for recognizing individuals at high risk of developing cancer (10). It has been concluded that somatic mutations or polymorphisms of the TGF-b receptor genes (*TGFBR1/2*) or SMAD genes lead to alterations to this pathway, which have an association with the development and progression of colorectal cancer (11). 

*SMAD7* (Mothers Against Decantaplegic Homolog 7) acts as a negative regulator of TGF-β signaling via various mechanisms both in the cytoplasm and in the nucleus (12,13). *SMAD7* promotes the anti-inflammatory action of the TGF-β signaling pathway (14). Although *SMAD7* over expression has been proved to have a role in the inhibition of TGF-b-mediated fibrosis, carcinogenesis, and inflammation (15), there is some ambiguous points about the underlying mechanism.

It is obvious that some factors such as allele frequencies or specific linkage disequilibrium structure may cause the risk alleles variation to CRC risk between populations, or special genetic and environmental backgrounds may alter the effect of the variants (16,17). Therefore, the studying of variations in different populations would be so informative to reveal the disease mechanism.

Recently, genome-wide studies have recognized the *SMAD7* gene (*18q21*) as an associated modest locus but highly significant increase in colorectal cancer risk (18,19). Regardless the complex role of *SMAD7* as an intracellular mediator of TGF-b type 1 receptor in cancer development, the relevance of several genetic variants such as single nucleotide polymorphism (SNP) of *SMAD7* with colorectal cancer has been proved by some studies (18,-). In this paper, we set out to investigate the role of one polymorphisms of *SMAD7*: rs2337104 on colorectal cancer risk based on hypothesis that there may be the association between that SNP and colorectal cancer in an Iranian population. Additionally, we used the clinical data to explore the correlation between them and mentioned SNP. Factors evaluated including age, sex, tumor site, tumor grade and stage. 

## Patients and Methods

**Study population**

In this study, 109 CRC cases and 109 healthy subjects were enrolled from October 2007 to January 2009 in cancer registry unit of the Gastroenterology and Liver Disease Research Center, Shahid Beheshti University of Medical Science, Tehran, Iran. All patients were diagnosed with colorectal cancer and histologically confirmed by positive colonoscopy and pathology results for colon or rectum malignant tumor. Data on age, sex, tumor histology and TNM stage were collected from CRC patients medical records and histopathology reports. The histological classification and pathological staging were determined on the criteria of the UICC Tumor-Node-Metastasis classification of malignant tumors (TNM) 6^th^ edition, 2002, colon and rectum (ICD-O C-18-C20). All participants signed an informed consent prior to participation in the study, and all healthy subjects and patients completed a self-administrated questionnaire. Besides, Ethic Committee of the Research Center approved all procedures for gastroenterology and liver diseases. The controls were randomly selected in the same time. The control group consisted of individuals without colonoscopy report for malignancy inflammatory ulcers or polyps and without any history of gastrointestinal defects in their families. If our control population showed colonoscopy report for malignancy inflammatory ulcers or polyps, they were excluded from the study. Besides, they have no families with the history of gastrointestinal defects. 

**DNA isolation and genotyping**

Genomic DNA was extracted from peripheral blood using a phenol-chloroform standard protocol (28). Agarose gel-electrophoresis was used to assay the quality of genomic DNA, and then the concentration quantitated by NanoDrop1000. In this study, rs2337104 polymorphism was genotyped using predesigned TaqMan SNP genotyping assays (C-438205-10; Applied Biosystems; Foster City, CA). The reaction was performed on an ABI 7500 Real Time PCR System (Applied Biosystems). The following conditions were operated during the polymerase chain reaction: 95°C for 10 minutes and 40 cycles of 92°C for 15 seconds and 60°C for 1 minute. SDS software version 1.3 (Applied Biosystems) was the analytical tool to identify individual genotypes.

**Statistical analysis**

Statistical package for social sciences (SPSS) software version 13 was applied to calculate statistical analysis. χ^2^ test compared distribution of the allele and genotype frequencies and also clinicopathological characteristics. Logistic regression analysis, which used for the adjustment of confounding variables such as age, gender and smoking, was carried out to calculate odds ratio (OR) and its 95% confidence intervals (95% CI). The calculation of differences in quantitative and qualitative demographic variables was performed using student’s *t*-test or a χ^2^ test, respectively. Data were considered significant if they have *P*-value less than 0.05 in all comparisons. 

## Results

**Characteristics of the samples**

Among colorectal cancer patients participated in this study, 62 (56.9%) were males and 47 (43.1%) were females, with a mean age of 60.31 years (standard deviation, SD ±11.76); comparing to our control group with 44 (40.4%) men and 65 (59.6%) women (mean age: 44.32 years, standard deviation, SD ±16.28). Since a higher mean age was observed among patients (*P*=0.015), logistic regression was applied to remove its confounding effect. Of all 3 characteristics of colorectal cancer cases mentioned in Table 1, age and gender associations generated *P*-values less than 0.05 (*P*<0.001 and *P*=0.015, respectively).

**Table 1:** Characteristics of colorectal cancer patient and control groups

Characteristics	Patients (n=109)*	Controls (n=109) †	P-Value
Age (years)			<0.001
Gender			0.015
Male	62 (56.9)‡	44(40.4)	
Female	47 (43.1)	65 (59.6)	
Smoking status			0.251
Never	90 (82.6)	96 (88.1)	
Current	19 (17.4)	13 (11.9)	

* Mean (SD)= 60.31±11.76;

† Mean (SD)= 44.32±16.28;

‡ Number (%)

Demographic characteristics of colorectal cancer patients are presented in Table 2. The majority of patient tumors were found to be well differentiated (45.9%). Of all 109 patients with colorectal cancer, 73.4% had tumor located at colon and most of them showed no sign of metastasis (87.2%).

**Genotypic and allelic frequencies**

**Figure 1:**
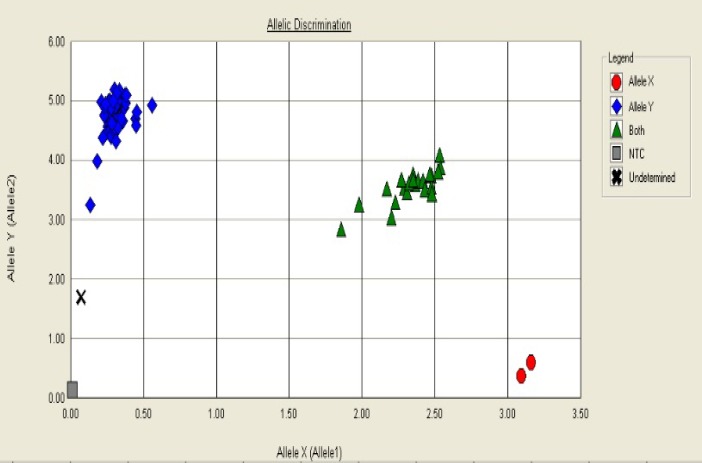
Allelic discrimination plot represents genotypes with four symbols (squares, diamonds, triangles, circles and multiplication sign) for NTC sample, TT, CT, CC, and undetermined genotypes respectively. The x-axis is amount of emission for flourophore channels (FAM) and on the y-axis represents emission for flourophore channels (VIC).

**Figure 2:**
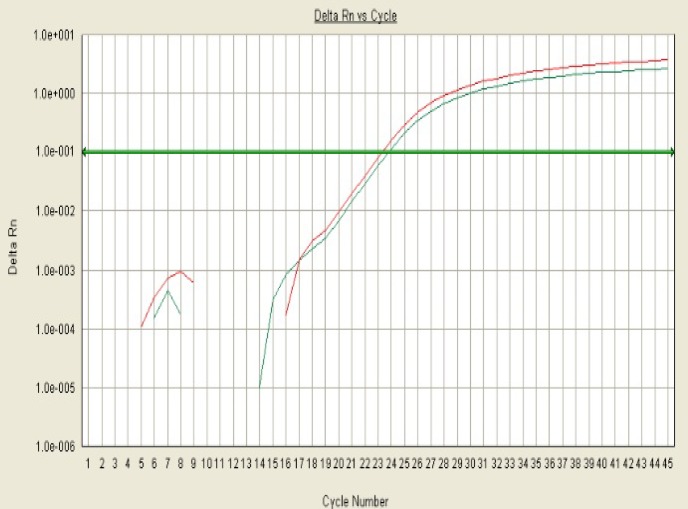
Allelic discrimination curves produced by the SDS analysis software. The x-axis is the amplification cycle number and on the y-axis represents raw fluorescent value. Example of a true heterozygote (CT) with the amplification curve for both flourophore channels (VIC, FAM).

Genotyping was performed by discrimination between the two alleles using analysis of the melting curves. Some representative allelic discrimination plots are given in Figures 1 and 2. 

rs2337104 genotypes were assessed for all patients, of which 82 (75.2%) were homozygous for TT genotype, 25 (22.9%) were heterozygous and 2 (1.8%) were homozygous for CC genotype. The frequencies of TT, TC, and CC genotypes among controls were 83.5%, 16.5%, and 0.0%, respectively (Table 3). The genotype distribution in our sample population conformed to Hardy-Weinberg equilibrium (*P*=0.347 for controls and *P*=0.953 for cases). These suggest that there was no population stratification and sampling bias.

**Table 2:** Characteristics of colorectal cancer patients

Variables	N	%
**Tumor grade**		
Well	50	45.9
Moderate	31	28.4
Poor	6	5.5
Not determined	22	20.2
**Location**		
Colon	80	73.4
Rctum	29	26.6
**TNM** * ** Stage**		
I+II	64	58.7
III+IV	45	41.3
**T**		
T1	2	1.8
T2	15	13.8
T3	77	70.6
T4	11	10.1
Unknown	4	3.7
**N**		
N0	61	56.0
N1	26	24.0
N2	11	10.0
Unknown	11	10.0
**M**		
M0	95	87.2
M1	14	12.8
**Duke’s stage**		
A	1	0.9
B	60	55.0
C	33	30.3
D	15	13.8

* TNM = Tumor Node Metastasis

A higher frequency of the homozygous genotype TT was observed in both patients and controls (75.2% and 83.5%, respectively), this genotype was defined as the reference. As revealed by these results, genotypic and allelic distribution of this genetic polymorphism of SMAD7 was not significantly different between CRC patients and controls. 

Although there was no statistically significant association between alleles and disorder, T allele was the most ones in this population and its frequency was 86.7% in patients compared with 91.7% in controls (OR=1.705, 95% CI=0.916-3.172; *P*=0.089).

**Table 3:** Distribution of SMAD7 genotypes among colorectal cancer patients and controls

	Genotype	Patients* (n=109)	Controls* (n=109 )	P_-_value†	OR (95% CI)	
					Crude	Adjusted‡
Rs2337104						
	TT	82 (75.2%)	91(83.5%)	0.313	1 (Reference)	1 (Reference)
	TC	25(22.9%)	18(16.5%)	0.127	1.541 (0.784-3.028)	1.866 (0.837-4.162)
	CC	2(1.8%)	0 (0.0%)	--	--	--
Allele frequencies						
	T	189(86.7%)	200 (91.7%)	0.089	1 (Reference)	
	C	29(13.3%)	18 (8.3%)		1.705 (0.916 -3.172)	

* The observed genotype distribution of patients and controls were in agreement with the Hardy-Weinberg equilibrium;

† *P*-values were for the difference in genotype frequencies between patients and controls;

‡ ORs were adjusted for age, gender and smoking status

Association between SMAD7 genotypes and clinicopathological characteristics were analyzed (Table 4). No significant correlation was found between the genotype distribution of rs2337104 polymorphism and any of the clinical and pathological (tumor localization, differentiation and metastasis), when stratified by gender, age and smoking status; hence revealing no association between polymorphic status and the risk of developing colorectal cancer in the studied population. 

## Discussion

As *SMAD7* has a crucial regulatory role in the TGF-b signaling pathway which strongly contributed to tumor initiation and development (29,30), the present study was organized to investigate the correlation between one polymorphism of *SMAD7* gene, rs2337104, and CRC risk in an Iranian population. Furthermore, the association of this polymorphism with clinicopathological factors such as tumor location, TNM stage and tumor grade was explored. The results of our analysis indicated that variant genotypes of this polymorphism had no association with the risk of colorectal cancer in our population.

Although *SMAD7* has been extensively studied, there are still many unresolved questions about the underlying mechanisms. The first point is that *SMAD7* may be differently regulated in various tumors depending on the context analyzed as it has the pro- and anti-tumorigenic effects in different cancer types (12). As another prominent issue, *SMAD7* promotes the anti-inflammatory action of the TGF-β signaling pathway (14), but it has other mechanisms that are relevant to CRC. For instance, according on the results of other studies, *SMAD7* degrades β-catenin signaling that alters the Wnt-signaling pathway which has a central role in CRC  (31). Along with these challenges, Boulay et al. reported that the deletion of Smad7 in CRC patients had a promising clinical outcome compared with patients with *SMAD7 *amplification  (31), and Halder et al. found that Smad7-overexpressing FET cells show aggressive colony formation on soft agar and increased tumorigenicity in vivo in comparison with control FET cells (32). Conversely, the opposing role of *SMAD7* in the control of sporadic and colitis-associated CRC has been shown by one study in which they reported that over-expression of *SMAD7* in T cells associates with severe colitis and reduces the growth of colitis-associated CRC (33). Therefore, it seems to need a further research on the functionality of *SMAD7 *variants to unravel these complexities about the observed associations.

The rs2337104 T/C polymorphism is located on intron3 of *SMAD7* gene. Sequence comparison of the intron 3 of *SMAD7 *of several vertebrate species revealed the presence of a number of highly conserved noncoding regions (HCNRs) in the area (26). Since there is no study in which the genotype distributions of SNP: rs2337104 has been reported, our discussion revolves around the genotype frequencies of other polymorphisms, on this location (intron3). According to GWAS reports (18,19), intron 3 includes some polymorphisms of *SMAD7* genes that they have a significant association with increased risk of CRC.

**Table 4:** Association between *SMAD7* genotypes and clinicopathological characteristics

Characteristics	Genotype			
	CC	CT	TT	P*-*value
**Tumor grade **				0.731
Well	2	9	39	
Moderate	0	8	23	
Poor	0	2	4	
Not determined	0	6	16	
**Location**				0.454
Colon	2	20	58	
Rectum	0	5	24	
**TNM** * ** Stage**				0.385
I+II	2	13	49	
III+IV	0	12	33	
**Dukes stage**				0.801
A	0	0	1	
B	2	12	46	
C	0	8	25	
D	0	5	10	
**T**				0.601
T1	0	0	2	
T2	1	5	9	
T3	1	15	61	
T4	0	3	8	
Unknown	0	2	2	
**N**				0.652
N0	2	13	46	
N1	0	7	19	
N2	0	1	10	
Unknown	0	4	7	
**M**				0.429
M0	2	20	73	
M1	0	5	9	

* TNM, Tumor Node Metastasis

Yanliang et al. and Slattery et al. separately showed that there is an association between rs12953717 polymorphism and increased risk for CRC (22,25). In this line, Xin et al. also found that this polymorphism has a significant association with the CRC in Chinese population (23). Conversely, Thompson et al. reported the evidence for the association of rs12953717 with CRC in women only (21). Another intronic polymorphism, rs4939827, association was investigated by several groups. Kirac et al. results showed association of rs4939827 with colorectal cancer risk in Croatian population (20). Qibin et al. and Garcia-Albeniz et al, findings were also consistent to the two earlier studies in which rs4939827 was relevant to CRC (27, 34). In contrast, Xin et al. study revealed there was no association between this polymorphism and CRC (23), and Thompson et al. also presented the evidence for the association of rs4939827 with CRC in women only (21). The possible hypothesis for interpretation of such inconsistent results is that the ethnicity should be the causal of these discrepancies.

During this study, we found T allele is the most frequent allele in our population. However, allele frequencies of this polymorphism did not show a significant association between patients and healthy groups. According on International Hap-MAP project, this finding is consistent to other population such as Northern and Western European ancestry from the CEPH collection (94.7%), Han Chinese in Beijing, China (97.6%), Japanese in Tokyo, Japan (94.2%), Mexican ancestry in Los Angeles, California (91.8%), but some other areas have lower frequency. For instance, with African ancestry in Southwest USA (63.2%), Maasai in Kinyawa, Kenya (41.5%), Yoruban in Ibadan, Nigeria (58.8%) (http://hapmap.ncbi.nlm.nih.gov/cgi-perl/gbrowse/hapmap28_B36). Geographic or ethnic variation and environmental factors should be the possible options for such discrepancies in allele frequencies in different population. Furthermore, *SMAD7* genotypes were not notably associated with clinicopathological characteristics. The association of polymorphisms and clinicopathological features for CRC has been shown by Mates et al. in 2012 (35). Their results demonstrated an association between rs2939827 and rs3802842 of *SMAD7* gene with the site-specific difference of CRC. They reported that carriers of risk alleles at these loci could increase the susceptibility to development of rectal cancer rather than colon cancer. C allele carriers at rs3802842 were associated with a lower risk for rectal tumors. Our results indicated that we couldn’t recommend the *SMAD7* gene to be associated with progression or metastasis of colorectal cancer in an Iranian population.

This study was conducted in well-defined homogenous samples with detailed clinical data. However, Sample size was our first limitation. It was difficult to gather samples with detailed clinical data during restricted time. Since the genotype differences may be strictly attributed to chance due to the modest sample size, larger population should be studied to clarify the exact conclusion of this SNP frequency in our population. Although the results showed no significant difference between genotypes of this polymorphism and CRC susceptibility**,** it could be significant with considering a significance level as 10%. Therefore, it is advisable that future studies with larger sample numbers be done. The second is that only one polymorphism of the *SMAD7* gene was studied, and it is not logical to conclude about the effect of whole gene on CRC development. 

In conclusion, this is the first case-control study to investigate the influence of rs2337104 T/C of *SMAD7* gene on clinicopathological features and CRC risk in Iranian population. According on our findings, there was no evidence of association this SNP and the risk of initiation and development of CRC and no significant effect of this SNP on clinicopathological features. To regard this salient point that the results have been gathered within the context of some unavoidable limitations such as small sample size; further studies in various populations should be implemented to elucidate the association of this SNP with colorectal cancer.
